# In Vitro Evaluation of Sugar-Conjugated Thienopyrimidinone Derivatives with Possible Neuroprotective and Antioxidant Effects

**DOI:** 10.3390/ijms262210826

**Published:** 2025-11-07

**Authors:** Asma K. Alshamari, Wael M. Aboulthana, Hayam Mansour, Khadiga M. Abu-Zied, Odeh A. O. Alshammari, Nesrin M. Morsy, Nuha O. S. Alsaif, Mona Z. Alshammari, Eman S. Nossier, Nasser A. Hassan

**Affiliations:** 1 Department of Chemistry, College of Science, Ha’il University, Ha’il 81451, Saudi Arabia; ak.alshamari@uoh.edu.sa (A.K.A.); odeh.alshammari@uoh.edu.sa (O.A.O.A.); n.alseif@uoh.edu.sa (N.O.S.A.); m.althonaian@uoh.edu.sa (M.Z.A.); 2 Biochemistry Department, Biotechnology Research Institute, National Research Centre, 33 El Buhouth Street, Cairo 12622, Egypt; wmkamel83@hotmail.com; 3 Department of Cell Biology, Biotechnology Research Institute, National Research Centre, 33 El Buhouth Street, Cairo 12622, Egypt; hayammansour@hotmail.com; 4 Department of Photochemistry (Synthetic Unit), Chemical Industries Research Institute, National Research Centre, 33 El Buhouth Street, Cairo 12622, Egypt; khashaheen@hotmail.com; 5 Organometallic and Organometalloid Chemistry Department, Chemical Industries Research Institute, National Research Centre, 33 El Buhouth Street, Cairo 12622, Egypt; nesrinmorsy@yahoo.com; 6 Department of Pharmaceutical Medicinal Chemistry and Drug Design, Faculty of Pharmacy (Girls), Al-Azhar University, Cairo 11754, Egypt; dr.emannossier@gmail.com

**Keywords:** neuroprotection, oxidative stress, neurodegeneration, multi-target agents, antioxidant activity, thieno [2,3-d]pyrimidine derivatives, glycosides, aldo-sugars, molecular docking

## Abstract

A series of glycosylated thienopyrimidinone derivatives (**7a–e** and **8a–e**), previously synthesized through a multi-step sequence involving a Gewald reaction, thiocyanate cyclization, functionalization with chloroacetic acid, and subsequent coupling with aldose sugars (glucose, mannose, galactose, xylose, and arabinose), were subjected to comprehensive biological evaluation. Structural confirmation of all compounds was achieved by spectroscopic and elemental analyses. Among them, compound **8e** displayed remarkable antioxidant capacity, with radical scavenging activity surpassing standard controls, and demonstrated significant neuroprotective potential through its ability to attenuate oxidative stress, a key driver of neurodegeneration. Furthermore, **8e** exhibited notable anti-arthritic and anti-diabetic effects, which may indirectly enhance neuroprotection by alleviating systemic inflammation and metabolic dysfunction—recognized risk factors for neurodegenerative disorders. Molecular docking and molecular dynamics studies revealed favorable binding interactions and structural stability of **8e** with multiple biological targets, supporting its promise as a multifunctional neuroprotective candidate against oxidative stress and neurodegeneration.

## 1. Introduction

Oxidative stress and chronic inflammation are key pathological processes involved in numerous chronic diseases, including arthritis, type 2 diabetes mellitus (T2DM), and neurodegenerative disorders such as Alzheimer’s disease (AD) [1,2,3]. Excessive generation of reactive oxygen species (ROS) triggers lipid peroxidation, protein denaturation, and activation of pro-inflammatory pathways [2,4], while abnormal glucose metabolism further aggravates oxidative and inflammatory damage [3,5]. In AD specifically, oxidative stress, dysfunctional glucose metabolism, and β-amyloid (Aβ) aggregation synergistically accelerate neurodegeneration [1]. Therefore, compounds that simultaneously scavenge ROS, inhibit inflammatory mediators, and modulate carbohydrate-metabolizing enzymes are of great therapeutic interest [6,7,8]. Arthritis is a progressive inflammatory disorder characterized by structural damage to joint proteins and heightened proteinase enzyme activity, both of which play a central role in tissue deterioration and discomfort. Targeting these enzymatic pathways offers a viable approach for therapeutic intervention in arthritic conditions [2].

**Figure 1:**
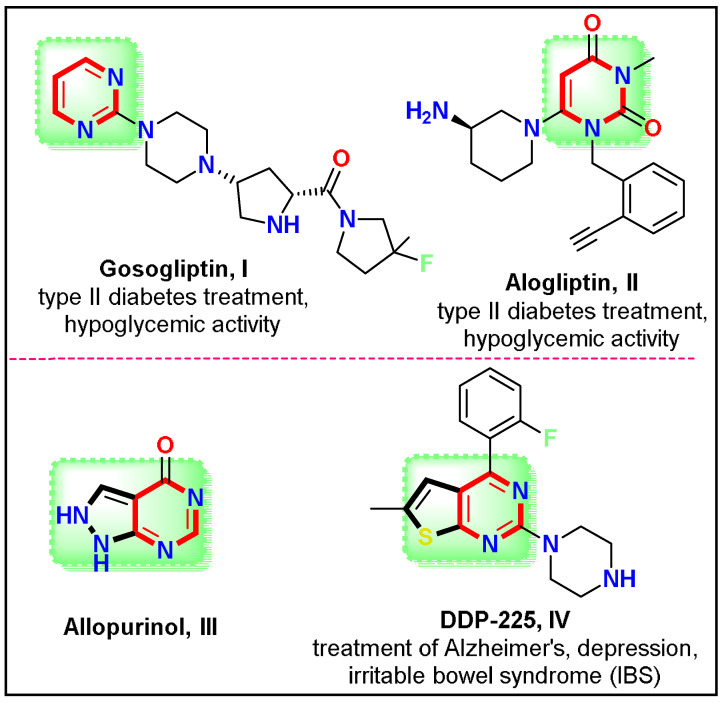
Marketed and clinically approved pyrimidine/fused pyrimidines, revealing antidiabetic, antioxidant, or anti-Alzheimer activity.

**Figure 2:**
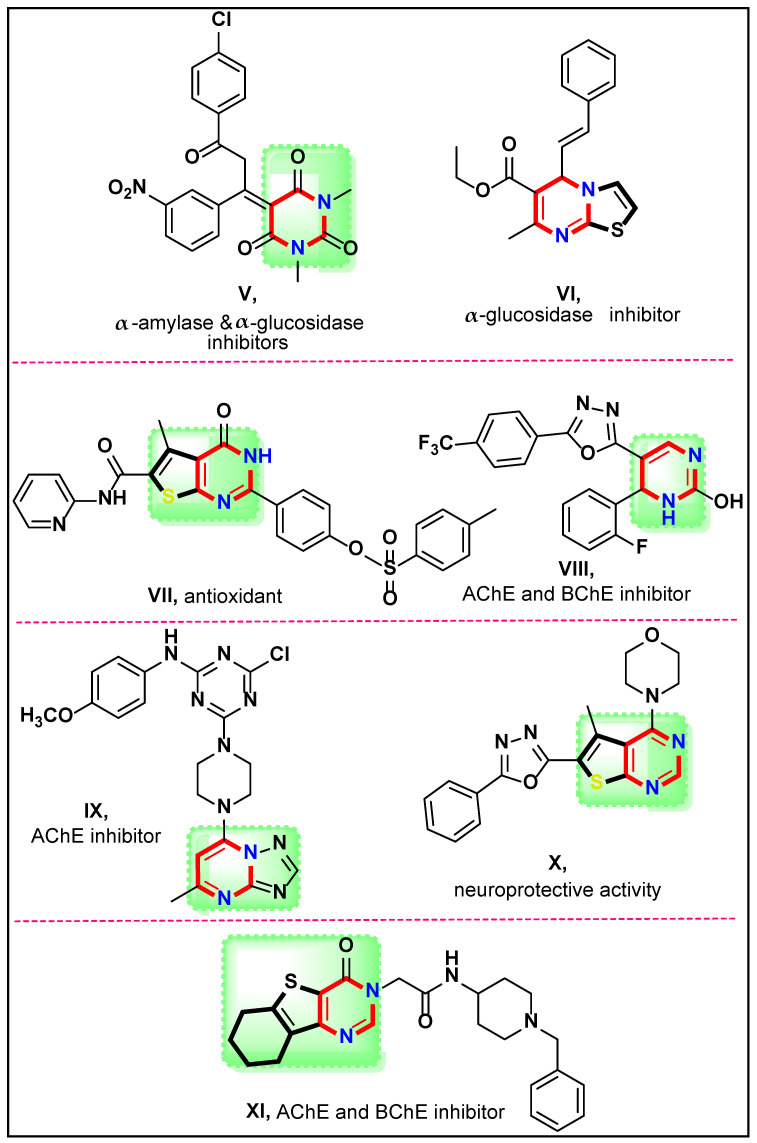
Reported pyrimidine/fused pyrimidines, demonstrating antidiabetic, antioxidant, or anti-Alzheimer activity through different mechanisms of action.

The design and synthesis of heterocyclic compounds with multifunctional pharmacological profiles remain at the forefront of medicinal chemistry. The most common members of the diazine family, pyrimidines, are biologically significant heterocycles that include uracil and thymine, which are components of ribonucleic acid (RNA) and deoxyribonucleic acid (DNA), as well as cytosine [9]. Many pyrimidines and fused pyrimidine analogs have been shown to possess antibacterial, antiviral, antifungal, antileishmanial, analgesic, anti-inflammatory, anti-Alzheimer’s, antipyretic, antihypertensive, antidiabetic, antiallergic, herbicidal, anticonvulsant, anticancer, and antihistaminic activities. Numerous derivatives of pyrimidines and fused pyrimidines are also reported to have potential central nervous system (CNS) depressant properties and act as calcium channel blockers [10,11,12,13,14]. Figure 1 and Figure 2 illustrate marketed and clinically approved drugs, and reported derivatives bearing pyrimidines and fused pyrimidines **I**–**XI**, that represented antidiabetic, antioxidant, or anti-Alzheimer activity through different mechanisms of action [15,16,17,18,19,20,21,22].

Figure 1 shows examples of marketed antidiabetic drugs with pyrimidine cores, including gosogliptin **I** and anagliptin **II**, which suppress dipeptidyl peptidase-IV (DPP-4) activity [15]. Additionally, fused pyrimidines e.g., pyrazolo [3,4-*d*]pyrimidine, allopurinol **III**, was intended to be used as antioxidant and thieno [2,3-*d*]pyrimidine, DDP-225 **IV**, described in the treatment of Alzheimer’s, irritable bowel syndrome (IBS), and depression [16,17].

Thieno [2,3-*d*]pyrimidines have gained significant attention due to their structural similarity to naturally occurring nucleotides and their diverse biological activities [23,24], including antioxidant as **VII** [19], anti-inflammatory, anti-diabetic, antimicrobial, and neuroprotective effects like **X** [17] (Figure 2). The π-rich, electron-deficient nature of this fused heterocycle facilitates fine-tuning of its biological interactions through rational substitutions, making it a promising framework for multi-target drug design. Recent studies have further demonstrated that thieno [2,3-*d*]pyrimidine derivatives possess diverse pharmacological activities relevant to multifactorial diseases such as AD. For example, Eissa et al. synthesized S-substituted tetrahydrobenzothienopyrimidine based on the donepezil scaffold **XI** and reported dual acetylcholinesterase (AChE) and butyrylcholinesterase (BuChE) inhibition, antioxidant activity, and suppression of Aβ aggregation—hallmarks of AD pathogenesis [25]. Derivative **X** has demonstrated neuroprotection against oxidative damage in PC12 cells [26]. Meanwhile, some analogues bearing this scaffold have exhibited potent dipeptidyl peptidase-IV (DPP-IV) inhibitory activity, suggesting potential anti-diabetic properties [27].

Moreover, thiazolidinediones (TZDs) are well-known PPARγ-modulating antidiabetic scaffolds, and recent work by Naim et al. demonstrated that 2,4-TZD amide derivatives exhibit enhanced glucose-lowering efficacy through optimized receptor interactions [28]. Similarly, Elmongy et al. reported the design of cycloheptathiophene/thienopyrimidine derivatives with potent free-radical scavenging activity (DPPH) surpassing ascorbic acid, highlighting the versatility of heterocycles in targeting both metabolic and oxidative stress-related pathways [29].

Glycosylation of heterocyclic compounds is a well-established strategy to improve solubility, membrane permeability, and biological specificity [30,31]. The introduction of sugar moieties can enhance hydrogen bonding, optimize drug–target interactions, and sometimes increase metabolic stability. Moreover, sugar-conjugated heterocycles have been reported to exhibit potent antioxidant and anti-inflammatory properties, as well as improved interactions with carbohydrate-metabolizing enzymes [32,33,34]. Building on our prior research in designing bioactive molecules [35,36,37,38,39,40,41,42,43,44,45,46,47,48], this study focuses on two previously synthesized series of sugar-conjugated thienopyrimidinone derivatives (**7a–e** and **8a–e**) and evaluates their antioxidant, potential neuroprotective, anti-arthritic, and anti-diabetic activities. Additionally, molecular docking and molecular dynamics simulations were employed to investigate the binding interactions and stability of selected compounds with biological targets relevant to these diseases.

## 2. Results and Discussion

### 2.1. Chemistry

**Scheme 1:**
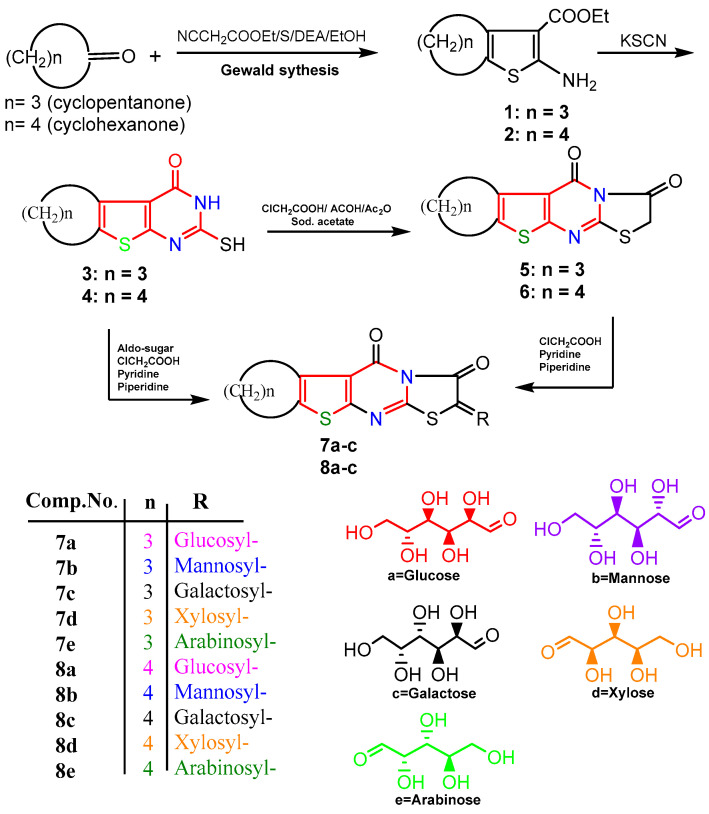
Synthesis of thienopyrimidinone glycosides **7a–e**, **8a–e**.

The synthesis of the target glycosylated thienopyrimidinone derivatives (**7a–e** and **8a–e**) has been previously reported by our group [45]. In brief, β-enamino esters **1** and **2** were prepared via a Gewald reaction of cyclopentanone or cyclohexanone with ethyl cyanoacetate and sulfur in the presence of diethylamine [49]. Subsequent treatment with potassium thiocyanate afforded the corresponding 2-mercapto-thienopyrimidinones **3** and **4**. These intermediates were further functionalized by condensation with chloroacetic acid to give the carboxymethyl derivatives **5** and **6**, which underwent glycosylation with various monosaccharides (glucose, mannose, galactose, xylose, arabinose) to furnish the final products **7a–e** and **8a–e**, as depicted in Scheme 1.

The resulting sugar-conjugated products (**7a–e** and **8a–e**) were purified by crystallization from appropriate solvents, and their identities were confirmed by comparison of melting points, elemental analysis, and spectroscopic data (IR, ^1^H NMR, ^13^C NMR) with our previously reported values. For example, compound **7b** displayed in its IR spectrum (KBr) characteristic absorption bands at 3434 cm^−1^ for hydroxyl (OH) groups, 2857 cm^−1^ for aliphatic C–H stretches, and 1666 and 1660 cm^−1^ corresponding to two carbonyl functionalities. The ^1^H NMR spectrum (500 MHz, DMSO-d_6_, δ ppm) showed signals at 2.30 (m, 2H, CH_2_), 2.49 (t, 2H, CH_2_), 2.50 (t, 2H, CH_2_), 3.05 (m, 1H, H-33.0 3.11 (m, 2H, H-611 H-611 3.40 (t, 1H, H-440 3.16 (m, 5H, OH protons, D_2_O exchangeable), 3.52 (m, 1H, H-2xch and 5.96 (d, 1H, H-1′, *J* = 7.5 Hz). The ^13^C NMR spectrum (100 MHz, DMSO-d_6_, δ ppm) revealed resonances at 24.9, 25.8, and 31.9 (CH_2_ groups); 63.3, 64.4, 70.9, 72.7, and 74.6 (sugar carbon atoms); 117.6 (C=C, pyrimidine); 125.4 (C=C–S, cyclopentyl ring); 126.0 (C=C–S, thiophene ring); 139.4 (S–C–N, cyclopentyl); 148.2 (sugar carbon atom); 155.5 (C=C, pyrimidine); 158.3 (N–C=N); 168.6 (C=O, pyrimidine); and 171.3 (N–C=O, thiophene ring). These structurally diverse glycosides combine the bioactive thienopyrimidinone scaffold with carbohydrate moieties, which may enhance their pharmacokinetic properties and biological activities. The current study focuses on evaluating these previously synthesized compounds for their antioxidant, neuroprotective, anti-arthritic, and anti-diabetic activities, supported by molecular docking analyses.

### 2.2. Biological Investigations

The sugar-conjugated thienopyrimidinone derivatives (compounds **7a–e** and **8a–e**) were subjected to a broad biological evaluation covering antioxidant, anti-diabetic, anti-arthritic, and anti-alzheimer activities. These compounds, originally reported in a prior publication for their chemical synthesis, were repurposed for biological investigation, showing promising multi-target potential.

#### 2.2.1. Antioxidant and Radical Scavenging Evaluation

Oxidative stress, caused by excessive reactive oxygen species (ROS) such as superoxide anion (O_2_^•−^), hydroxyl radical (OH^•^), nitric oxide (NO^•^), and hydrogen peroxide (H_2_O_2_), is implicated in cancer, cardiovascular disease, diabetes, and neurodegeneration. Neutralizing these species with natural or synthetic antioxidants helps maintain redox balance and protect biomolecules [50,51,52,53,54]. Nitrogen- and sulfur-containing heterocycles often display strong antioxidant activity through electron or hydrogen donation, metal chelation, and radical stabilization. The antioxidant potential of the synthesized derivatives was assessed by total antioxidant capacity (TAC), iron-reducing power (IRP), and radical scavenging assays (DPPH, ABTS, NO, OH, and H_2_O_2_), with ascorbic acid serving as the reference standard. The TAC and IRP results are summarized in Table 1.

Among the tested compounds, **8b** and **8e** emerged as the most potent antioxidants. Compound **8b** exhibited a TAC of 68.01 mg gallic acid/g and an IRP of 60.86 µg/mL, while compound **8e** showed slightly higher values, with a TAC of 68.69 mg gallic acid/g and an IRP of 61.54 µg/mL. These results are comparable to those of the standard antioxidant, ascorbic acid, which demonstrated a TAC of 78.44 mg/g and an IRP of 67.49 µg/mL.

In contrast, compounds from the **7a–e** series demonstrated moderate to low antioxidant activity. For example, compound **7a** showed a TAC of 17.44 mg/g and an IRP of 10.29 µg/mL. Its IC_50_ values were significantly higher, exceeding 27 µg/mL for DPPH and surpassing 48 µg/mL for both OH and H_2_O_2_ scavenging assays. These findings underscore the influence of structural differences between the **7a–e** and **8a–e** series on antioxidant performance, with the latter exhibiting superior activity likely due to enhanced electronic and steric properties.

The radical scavenging efficiency of compound **8e** was further confirmed by its low median inhibitory concentrations (IC_50_) across all tested radicals, as presented in Table 2. Specifically, **8e** recorded IC_50_ values of 5.95 µg/mL for DPPH, 4.73 µg/mL for ABTS, 6.80 µg/mL for nitric oxide (NO), 9.14 µg/mL for hydroxyl radical (OH), and 10.17 µg/mL for hydrogen peroxide (H_2_O_2_). These values closely approximate those of ascorbic acid, which showed IC_50_ values of 5.45 µg/mL (DPPH), 4.35 µg/mL (ABTS), 6.18 µg/mL (NO), 8.31 µg/mL (OH), and 9.24 µg/mL (H_2_O_2_).

**Figure 3:**
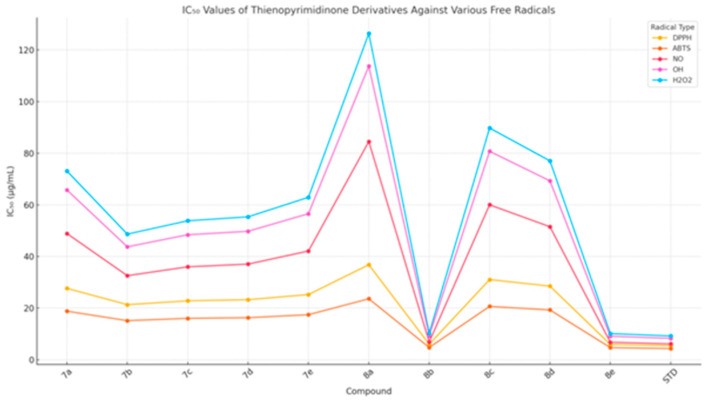
IC_50_ values (µg/mL) of thienopyrimidinone derivatives (**7a–e**, **8a–e**) against free radicals. Data shown as means. STO: Standard antioxidant.

Notably, both compounds **8b** and **8e** exhibited greater than 65% inhibition at a concentration of 20 µg/mL across all radical assays, indicating broad-spectrum antioxidant efficacy. These comparative inhibitory profiles are illustrated in Figure 3.

##### Structure–Activity Relationship (SAR) and Rationalization

The antioxidant activity of the synthesized thienopyrimidinone derivatives (**7a–e** and **8a**–**e**), as presented in Table 1 and Table 2 and illustrated in Figure 3, reveals a clear structure–activity relationship (SAR), governed primarily by the nature of the sugar moiety conjugated at position 2 and the size of the fused ring system.

The sugar units attached to the thiazolo [3,2-a]pyrimidine-3,5(2H)-dione scaffold play a pivotal role in modulating antioxidant performance. Compound **8b**, which incorporates a mannosyl group bearing five hydroxyl groups, demonstrated enhanced hydrogen atom transfer (HAT) capacity and superior radical stabilization, resulting in notably low IC_50_ values across all assays. In comparison, compound **8e** contains an arabinosyl moiety with four hydroxyl groups, offering slightly fewer hydrogen-donating sites but still exhibiting potent antioxidant activity. These findings suggest that hydroxyl group density directly influences the compound’s ability to neutralize reactive oxygen species (ROS).

Another critical determinant of antioxidant potency is the nature of the fused ring system. The **7a–e** series features a cyclopentane-fused scaffold, which imparts limited conformational flexibility and moderate antioxidant activity. In contrast, the **8a–e** series incorporates a cyclohexane-fused core, offering greater spatial adaptability and more favorable orientation of substituents. This structural advantage facilitates improved interaction with ROS, contributing to the consistently superior antioxidant performance observed in the **8a–e** compounds.

Taken together, the combination of a hydroxyl-rich sugar moiety and a cyclohexane-fused thienopyrimidinone backbone significantly enhances antioxidant efficacy. This synergy is reflected in the low IC_50_ values recorded across multiple radical scavenging assays, underscoring the therapeutic potential of these compounds as multifunctional antioxidant agents.

#### 2.2.2. Anti-Alzheimer Activity (AChE Inhibition)

Alzheimer’s disease is characterized by progressive neurodegeneration, with acetylcholinesterase (AChE) playing a central role in its pathology. Inhibiting AChE is a well-established therapeutic strategy, as it enhances cholinergic neurotransmission by preventing the breakdown of acetylcholine, thereby improving cognitive function [55].

The anti-Alzheimer’s potential of the synthesized sugar-conjugated thienopyrimidinone derivatives (**7a–e** and **8a–e**) was evaluated through AChE inhibition. As shown in Table 3, all compounds exhibited modest, concentration-dependent inhibition at 20 µg/mL, with activities ranging from 4.11% (**7e**) to 5.93% (**8c**). These effects were markedly weaker than the reference drug Donepezil, which achieved 54.46% inhibition with an IC_50_ of 5.26 µg/mL, highlighting its superior potency.

Despite this disparity, compound **8c** emerged as the most active analog within the synthesized series, achieving the highest AChE inhibition (5.93 ± 0.19%) and the lowest IC_50_ (48.46 ± 1.91 µg/mL) among all tested compounds. While its absolute potency remains limited, its relative performance highlights it as a potential lead scaffold for further structural refinement.

The modest activity observed across the series suggests that the current scaffolds may not yet possess optimal pharmacophores for cholinesterase inhibition. However, the incorporation of sugar moieties and fused ring systems in the **8**-series appears to enhance AChE interaction compared to the **7**-series, indicating a promising direction for future analog development. These findings support the continued exploration of sugar-conjugated thienopyrimidinones as multi-target agents, where AChE inhibition may complement broader antioxidant and anti-inflammatory effects.

#### 2.2.3. Anti-Arthritic Activity

Arthritis is a multifactorial condition marked by joint inflammation, pain, and progressive tissue degeneration. Inflammatory mechanisms, particularly protein denaturation and proteinase activity, are central to the pathogenesis of rheumatoid arthritis (RA). In this study, the anti-arthritic potential of the synthesized thienopyrimidinone derivatives was assessed using two well-established in vitro models: protein denaturation and proteinase inhibition assays [56,57,58].

As shown in Table 3, the anti-arthritic potential of the synthesized compounds was evaluated using protein denaturation and proteinase inhibition assays, two established models for assessing anti-inflammatory. Most derivatives displayed only modest inhibition in the protein denaturation assay (8–15%); however, compound **8e** exhibited a remarkable 54.07 ± 0.65% inhibition, closely approaching the activity of Diclofenac sodium (60.74 ± 0.14%). A comparable trend was observed in the proteinase inhibition assay, where 8e again demonstrated strong activity (50.90 ± 0.65%), while Diclofenac sodium achieved 56.57 ± 0.14% inhibition and the remaining compounds showed considerably weaker effects (4–11%). Consistent with these findings, **8e** also recorded the lowest IC_50_ value (9.08 ± 0.11 µg/mL), indicating potent anti-arthritic activity nearly equivalent to that of diclofenac sodium (8.20 ± 0.06 µg/mL).

The remaining compounds exhibited higher IC_50_ values (42–102 µg/mL), reflecting weaker efficacy. Other compounds exhibited moderate activity, with inhibition percentages ranging from 8.74% (**8a**) to 15.06% (**7b**) for protein denaturation, and 4.57% (**8a**) to 10.89% (**7b**) for proteinase inhibition. These results suggest that structural features such as sugar conjugation and electron-rich substituents play a critical role in modulating anti-arthritic efficacy.

##### Structure–Activity Relationship (SAR)

The outstanding dual anti-arthritic activity of compound **8e** highlights the critical contribution of its sugar moiety—specifically, the arabinosyl substitution with four hydroxyl groups likely enhances hydrogen bonding and interactions with inflammatory protein targets, stabilizing the compound–protein complex. In contrast, the mannosyl-substituted derivative **8b**, despite also bearing multiple hydroxyl groups, exhibited only moderate inhibition. This suggests that the spatial orientation and steric accessibility of hydroxyl groups significantly influence activity, beyond their mere presence. Supporting this, studies have shown that chemical orientation of hydroxyls in carbohydrate moieties can markedly affect molecular interactions and biological efficacy [59,60]. Additionally, structural investigations in glycomimetic drug design have demonstrated how glycosidic linkage stereochemistry and sugar configuration directly modulate binding affinity and pharmacological behavior [61]. Furthermore, while the **7**-series compounds proved effective as acetylcholinesterase inhibitors, they displayed relatively weak anti-arthritic activity. This outcome underlines the idea that scaffold substitution patterns in this series favor cholinesterase inhibition rather than anti-inflammatory effects. Taken together, these findings underscore the influence of sugar configuration and hydroxyl orientation in fine-tuning the multi-target pharmacological profile of thienopyrimidinone derivatives.

#### 2.2.4. Anti-Diabetic Potential

Diabetes mellitus (DM) is a chronic metabolic disorder characterized by elevated blood glucose levels [62]. Two key enzymes, α-amylase and α-glucosidase, regulate carbohydrate digestion by breaking down polysaccharides into disaccharides and subsequently into monosaccharides. Inhibition of these enzymes is a well-established therapeutic strategy for controlling postprandial hyperglycemia in type 2 diabetes [63].

In this study, the anti-diabetic potential of the synthesized sugar-conjugated thienopyrimidinone derivatives (**7a–e** and **8a–e**) was assessed by evaluating their ability to inhibit α-amylase and α-glucosidase as depicted in Table 4. However, none of the tested compounds demonstrated significant inhibition of either enzyme at the examined concentrations (250, 500, and 1000 µg/mL).

At a concentration of 20 µg/mL, compounds **8b** and **8e** demonstrated exceptional dual inhibitory activity. Specifically, **8b** exhibited 61.53 ± 0.74% inhibition of α-amylase (IC_50_ = 6.78 ± 0.02 µg/mL) and 54.78 ± 0.74% inhibition of α-glucosidase (IC_50_ = 3.85 ± 0.05 µg/mL), while **8e** showed 62.18 ± 0.75% inhibition of α-amylase (IC_50_ = 6.71 ± 0.02 µg/mL) and 55.43 ± 0.75% inhibition of α-glucosidase (IC_50_ = 3.81 ± 0.05 µg/mL). These results are comparable to the standard drug Acarbose, which demonstrated 69.85 ± 0.16% and 63.10 ± 0.16% inhibition of α-amylase and α-glucosidase, respectively, with IC_50_ values of 5.98 ± 0.05 µg/mL and 3.34 ± 0.05 µg/mL.

In contrast, the **7a–e** series exhibited significantly lower activity, with α-amylase inhibition ranging from 13.37% to 17.32% and α-glucosidase inhibition between 24.10% and 31.25%. Their IC_50_ values were correspondingly higher, indicating weaker efficacy and limited therapeutic potential in this context.

##### Structure–Activity Relationship (SAR) Observations

The structure–activity relationship (SAR) analysis highlights the importance of both the fused ring system and the sugar moiety at position 2 in modulating anti-diabetic activity. The superior performance of **8b** and **8e** is attributed to their mannosyl and arabinosyl substitutions, respectively. These sugar units, rich in hydroxyl groups, likely enhance hydrogen bonding interactions with the active sites of α-amylase and α-glucosidase, thereby improving inhibitory potency. In contrast, the **7**-series compounds, which feature a cyclopentane-fused ring system, showed limited activity—suggesting that reduced conformational flexibility and suboptimal substituent orientation hinder effective enzyme binding. Among the 8-series, derivatives with fewer hydroxyl groups or less favorable sugar orientation (e.g., **8a**, **8c**, **8d**) exhibited moderate to low inhibition, reinforcing the importance of hydroxyl density and spatial arrangement for optimal activity.

Mechanistically, the observed enzyme inhibition is likely mediated through competitive binding at the catalytic sites of α-amylase and α-glucosidase. The sugar-conjugated derivatives mimic the natural substrates of these enzymes, allowing them to interfere with carbohydrate breakdown. The enhanced activity of **8b** and **8e** may result from a combination of factors: (i) hydrogen bonding between hydroxyl groups and key amino acid residues in the enzyme active site; (ii) steric complementarity provided by the cyclohexane-fused scaffold, facilitating optimal alignment; and (iii) an electron-rich heterocyclic core, contributing to π–π interactions and stabilization of the enzyme–inhibitor complex.

Taken together, these findings suggest that **8b** and **8e** are promising candidates for further development as multifunctional anti-diabetic agents, offering potent inhibition of carbohydrate-metabolizing enzymes with structural features that support strong and selective binding.

#### 2.2.5. Comparative Overview of Biological Profiles

##### Interrelationship Among Antioxidant, Anti-Alzheimer’s, Anti-Arthritic, and Anti-Diabetic Activities

**Figure 4:**
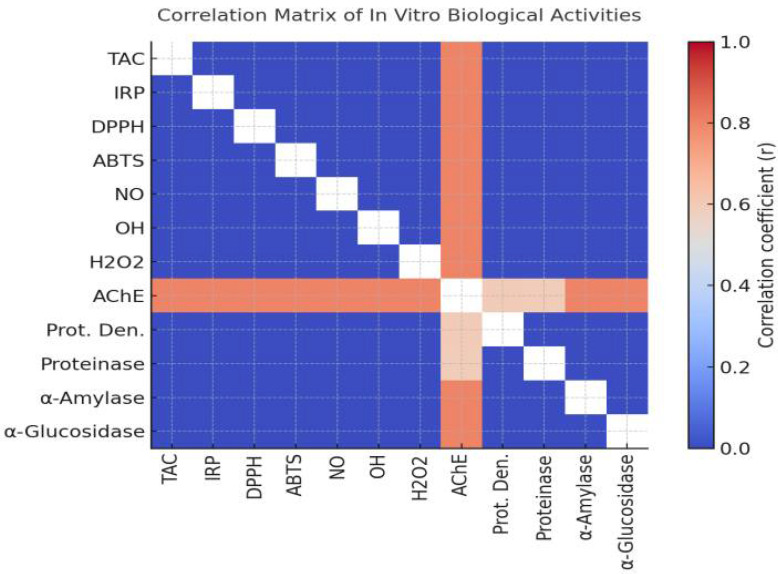
Correlation matrix of in vitro biological activities, showing correlation. coefficients (r) among antioxidant, anti-Alzheimer’s, anti-arthritic, and anti-diabetic assays.

To elucidate the mechanistic links among the investigated biological activities, a correlation analysis was performed using inhibition percentages at a fixed concentration (20 µg/mL) (Figure 4). The results revealed statistically significant and consistent positive correlations (*p* ≤ 0.01) between antioxidant parameters—total antioxidant capacity (TAC), iron-reducing power (IRP), and radical scavenging activities (DPPH, ABTS, NO, OH, and H_2_O_2_)—and all other tested bioactivities, including anti-Alzheimer (AChE inhibition), anti-arthritic (protein denaturation and proteinase inhibition), and anti-diabetic (α-amylase and α-glucosidase inhibition) effects.

Notably, high correlation coefficients (r ≈ 0.800) were observed between antioxidant indices and AChE inhibition, suggesting that the neuroprotective effects of the synthesized compounds may be partly mediated by their ability to mitigate oxidative stress. This aligns with established evidence implicating oxidative damage in cholinergic neuronal loss and synaptic dysfunction in Alzheimer’s disease [1].

Similarly, antioxidant activity correlated strongly with α-amylase and α-glucosidase inhibition, indicating a mechanistic link between redox modulation and glycemic control. This observation supports previous findings that oxidative stress contributes to the pathogenesis of type 2 diabetes mellitus [64].

The correlation between antioxidant and anti-arthritic activities—specifically protein denaturation and proteinase inhibition—further reinforces the hypothesis that inflammation and oxidative stress are interdependent processes in arthritis pathophysiology [65]. Moreover, a moderate yet significant correlation (r = 0.594–0.590) between AChE inhibition and anti-arthritic indices suggests overlapping inflammatory and oxidative pathways in neurodegenerative and autoimmune conditions.

Collectively, these findings indicate that the antioxidant potential of the synthesized compounds may underlie or synergize with their anti-Alzheimer, anti-diabetic, and anti-inflammatory properties. The observed interrelationships underscore the therapeutic promise of these molecules as multifunctional agents capable of targeting interconnected pathological processes.

These correlations also complement the structure–activity relationship (SAR) findings, which highlighted the role of specific sugar moieties and fused ring systems in modulating biological activity. Taken together, the SAR insights and correlation data provide a compelling rationale for further optimization of these scaffolds—not only as enzyme inhibitors but as broad-spectrum agents with multi-target therapeutic potential.

### 2.3. Molecular Docking Simulation

Docking simulations were performed to rationalize the in vitro inhibitory profiles of thieno [2,3-d]thiazolo [3,2-a]pyrimidine glycosides **8b** and **8e** against α-amylase and α-glucosidase.

#### 2.3.1. Docking Protocol Validation

First, acarbose, the native ligand (PDB codes: 1B2Y and 3WY1, respectively) [66,67], was re-docked into the active sites of α-amylase and α-glucosidase to validate the docking procedure. The resulting binding energies (−10.34 and −10.68 kcal/mol) and low RMSD values (1.22 and 0.79 Å) confirmed the reliability of the docking protocol.

#### 2.3.2. Docking of Compounds **8b** and **8e** with α-Amylase

**Figure 5:**
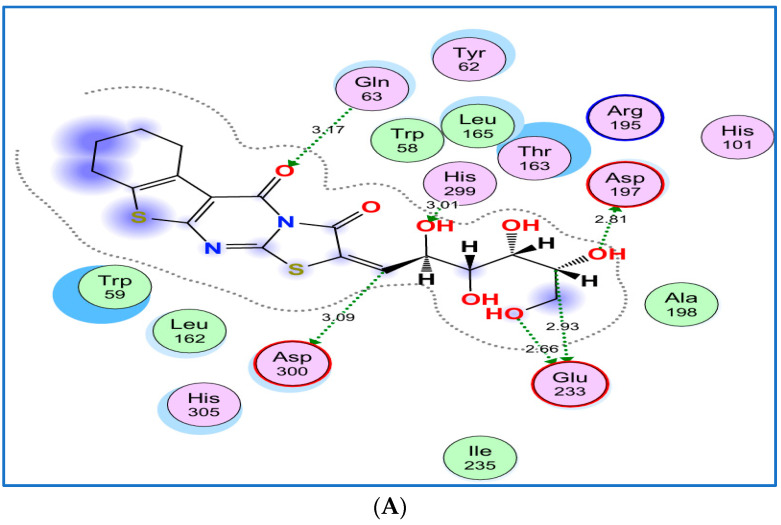

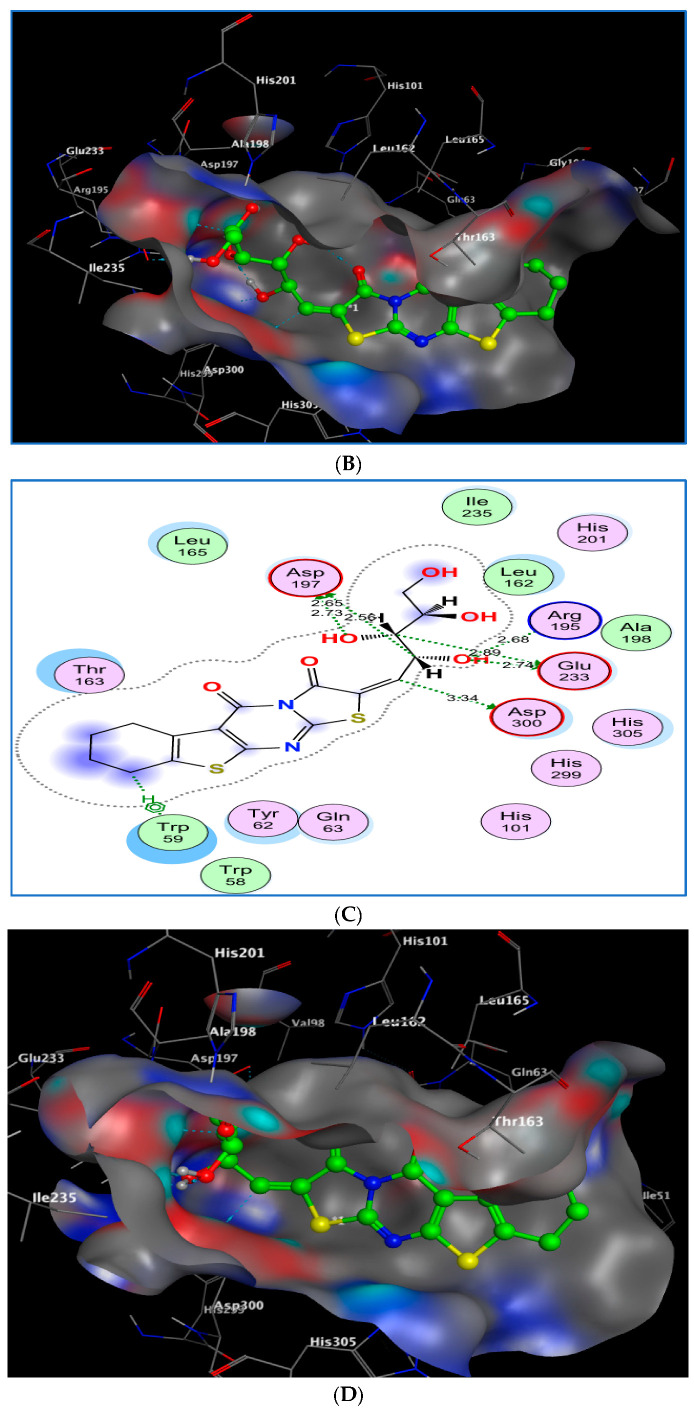
Binding interactions (2D and 3D views) of compounds **8b** and **8e** within the α-amylase active site (PDB code: 1B2Y) were depicted in (**A**–**D**) views, respectively.

After removal of the co-crystallized ligand, the screened thieno [2,3-d]thiazolo [3,2-a]pyrimidine glycosides **8b** and **8e** were docked into the α-amylase active site, producing binding energies of −10.52 and −10.38 kcal/mol, respectively. In both derivatives, the glycosyl moiety (mannosyl in **8b** and arabinosyl in **8e**) formed hydrogen bonds with key catalytic residues Asp197, Glu233, and Asp300, as well as with His299 in **8b** and Arg195 in **8e**. Additionally, in **8b**, Gln63 established an H-bond with the pyrimidinone carbonyl oxygen (distance: 3.17 Å). Hydrophobic contact was also observed, notably between Trp59 and the benzo [4,5]thieno [2,3-*d*]thiazolo [3,2-*a*]pyrimidine scaffold of **8e** as an arene-H interaction (Figure 5).

#### 2.3.3. Docking of Compounds **8b** and **8e** with α-Glucosidase

**Figure 6:**
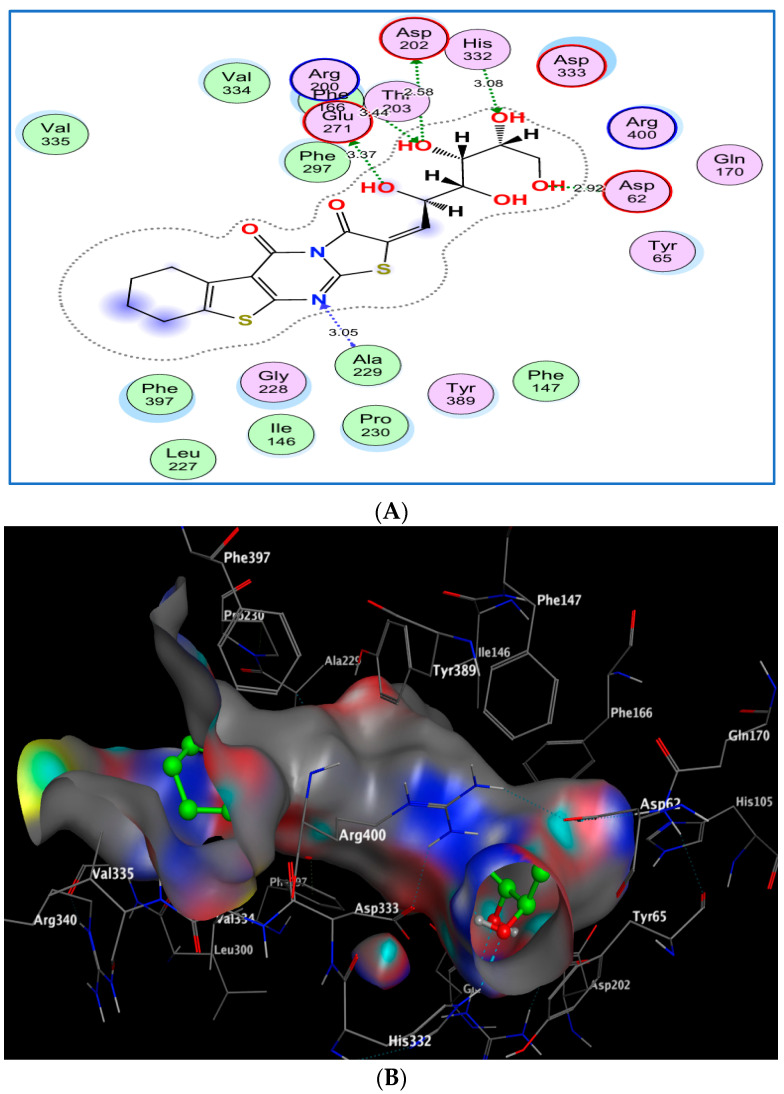

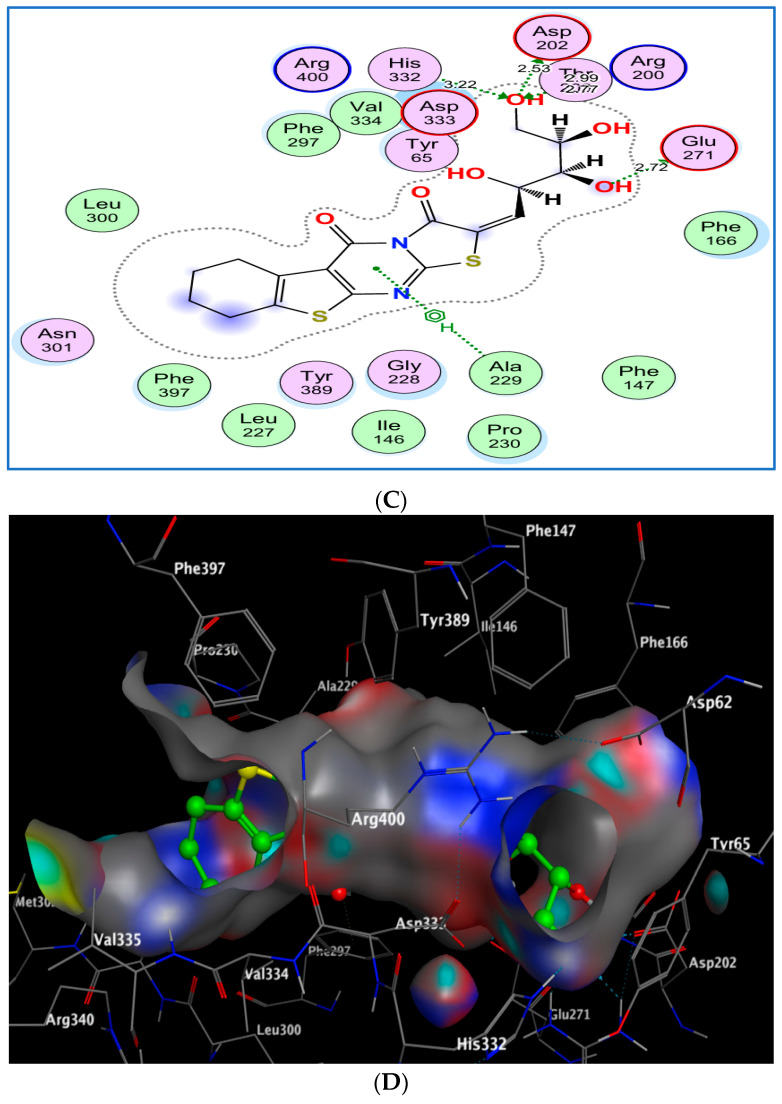
Binding interactions (2D and 3D views) of compounds **8b** and **8e** within the α-glucosidase active site (PDB code: 3WY1). were depicted in (**A**–**D**) views, respectively.

Regarding α-glucosidase, docking results revealed binding energies of –10.80 and –11.25 kcal/mol for **8b** and **8e**, respectively. Both derivatives exhibited hydrogen bonding between their glycosyl moieties and Arg200, Asp202, Glu271, and His332. In **8e**, an additional H-bond was detected with Asp62 (distance: 2.92 Å). The pyrimidine scaffold shared an interaction with Ala229, as an H-bonding with its nitrogen in **8b** (distance: 3.05 Å), and arene-H in **8e** (Figure 6).

#### 2.3.4. Interaction Summary and Comparative Insight

In agreement with previous reports, thieno [2,3-d]thiazolo [3,2-a]pyrimidine glycosides **8b** and **8e**, similar to acarbose, established multiple hydrophobic and hydrogen bonding interactions with critical residues (Asp197, Glu233, Asp300 in α-amylase and Arg200, Asp202, Ala229, Glu271, and His332 in α-glucosidase). These interactions contributed to their strong binding affinity and correspondingly significant inhibitory activity, as illustrated in Figure 6, which shows the 2D and 3D binding interactions of compounds 8b and 8e within the α-glucosidase active site.

### 2.4. Molecular Dynamics and System Stability

Molecular dynamics (MD) simulations were employed to predict the binding efficacy of the extracted compounds at the protein’s active site and to evaluate the stability and nature of their interactions over time [68,69]. Ensuring system stability is crucial for identifying anomalous motions and preventing simulation artifacts. To this end, the Root-Mean-Square Deviation (RMSD) was calculated over the 350 ns simulation trajectory to assess the structural stability of the protein-ligand complexes.

**Figure 7:**
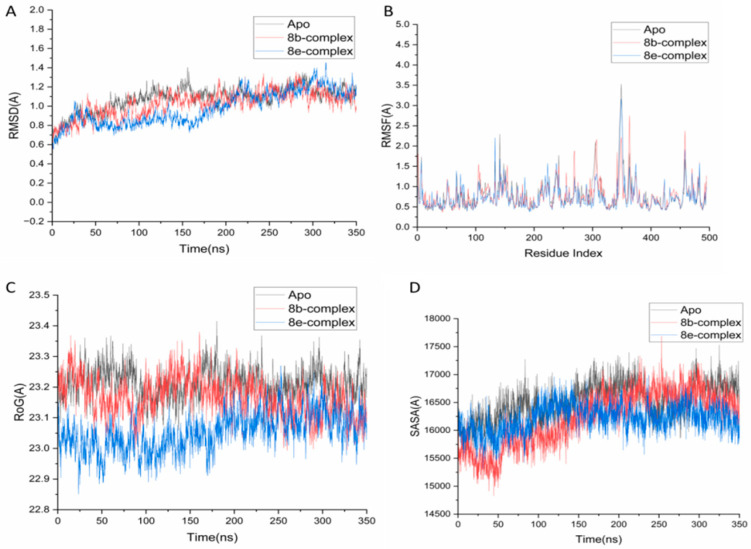
(**A**) RMSD of Cα atoms of the protein backbone atoms. (**B**) RMSF of each residue of the protein backbone Cα atoms of protein residues (**C**) ROG of Cα atoms of protein residues; (**D**) Solvent accessible surface area (SASA) of the Cα of the backbone atoms relative (black) to the starting minimized over 350 ns for the ATP binding site of amylase receptor with compound **8b** (red), and compound **8e** (blue).

**Figure 8:**
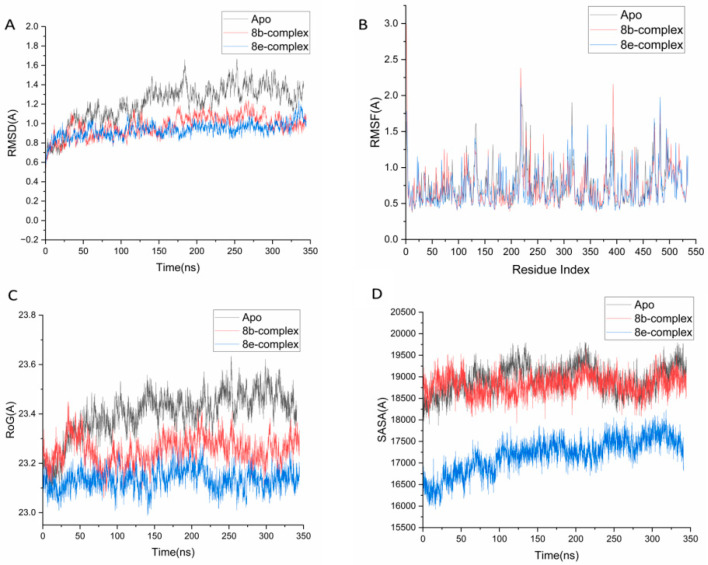
(**A**) RMSD of Cα atoms of the protein backbone atoms. (**B**) RMSF of each residue of the protein backbone Cα atoms of protein residues (**C**) ROG of Cα atoms of protein residues; (**D**) Solvent accessible surface area (SASA) of the Cα of the backbone atoms relative (black) to the starting minimized over 350 ns for the ATP binding site of glycosidase receptor with compound **8b** (red), and compound **8e** (blue).

The average RMSD values (Figure 7A) were 1.08 ± 0.12 Å for the Apo-amylase, 1.03 ± 0.12 Å for the **8b**-amylase complex, and 1.00 ± 0.18 Å for the 8e-amylase complex. Similarly, for the glycosidase systems (Figure 8A), the values were 1.21 ± 0.19 Å (Apo), 0.98 ± 0.10 Å (**8b**-complex), and 0.93 ± 0.07 Å (8e-complex). These results indicate that the 8e-protein complexes attained the most stable conformational state among all systems studied.

#### 2.4.1. Protein Flexibility

Protein flexibility upon ligand binding was investigated using Root-Mean-Square Fluctuation (RMSF) to analyze per-residue dynamics and their relationship with the bound ligand [70]. The average RMSF values (Figure 7B) were 0.79 ± 0.38 Å (Apo-amylase), 0.78 ± 0.33 Å (8b-amylase), and 0.74 ± 0.33 Å (8e-amylase). For the glycosidase targets (Figure 8B), the values were 0.76 ± 0.27 Å (Apo), 0.72 ± 0.28 Å (8b-complex), and 0.70 ± 0.25 Å (8e-complex). This data suggests that binding to compound **8e** effectively reduced residual fluctuations in both target proteins.

#### 2.4.2. Structural Compactness

The Radius of Gyration (Rg) was measured to evaluate the overall compactness and structural stability of the systems in response to ligand binding [71,72]. The average Rg values (Figure 7C) were 23.20 ± 0.05 Å (Apo-amylase), 23.26 ± 0.06 Å (8b-amylase), and 23.00 ± 0.06 Å (8e-amylase). For the glycosidase systems (Figure 8C), the values were 23.40 ± 0.09 Å (Apo), 23.25 ± 0.05 Å (8b-complex), and 23.13 ± 0.04 Å (8e-complex), indicating that the 8e complex exhibits a more rigid structure.

#### 2.4.3. Solvent Accessibility

Finally, the Solvent Accessible Surface Area (SASA) was quantified to probe the density of the hydrophobic core, a key factor in biomolecular stability [73]. The average SASA values (Figure 7D) were 16,528.70 Å^2^ (Apo-amylase), 16,256.87 Å^2^ (8b-amylase), and 16,200.14 Å^2^ (8e-amylase). The corresponding values for the glycosidase systems (Figure 8D) were 18,941.26 Å^2^ (Apo), 18,815.64 Å^2^ (8b-complex), and 17,188.06 Å^2^ (8e-complex). Collectively, the SASA findings, consistent with the RMSD, RMSF, and Rg analyses, confirm that the compound **8e** complexes remain stable and intact within the catalytic binding site of the target receptors.

## 3. Materials and Methods

### 3.1. Chemistry

All melting points were measured on a Gallenkamp melting point apparatus and are uncorrected. ^1^H-NMR (500 MHz) and ^13^C-NMR (100 MHz) spectra were recorded on a Varian spectrometer using DMSO-*d*_6_ as a solvent and TMS as an internal standard. Chemical shifts are reported in ppm. Coupling constants (*J*) are expressed in Hz. Mass spectra were recorded on a Varian MAT 112 spectrometer at 70 elV. Elemental analyses were performed at the Micro Analytical Centre, Cairo University, Egypt. Progress of the reactions was monitored by thin-layer chromatography (TLC) using aluminum sheets coated with silica gel F254 (Merck), viewing under a short-wavelength UV lamp effected detection. All evaporations were carried out under reduced pressure at 40 °C. The starting materials **2**, **3**, **4**, **5** and **6** were synthesized according to the references [45,46,47,48,49].

**General Procedure for the Synthesis of Glycoside Derivatives 7, 8 (a–e)** [45]

*Method 1:* As a one pot reaction a solution of compound **3** or **4** (10 mmole) in 30 mL pyridine was added to the proper aldo-sugars (10 mmole) and they were allowed to be heated under reflux for 8 h. After this came the addition of chloroacetic acid (10 mmole, 0.93 gm) and a few drops of piperidine. The reaction was monitored by using TLC technique and the reaction was stopped after completion. The reaction mixture was poured onto ice-water, and the precipitate was collected and recrystallized from the proper solvent to produce compounds **7a–e** and **8a–e**, respectively.

*Method 2:* A mixture of acetic anhydride, acetic acid, chloroacetic acid and anhydrous sodium acetate was added to (10 mmole) of compounds **3** and **4** then refluxed for 4 h, poured into cooled water and the collected precipitate was crystallized from ethanol to give brown precipitate with 70% yield of compounds **5** and **6**. Aldo-sugars (10 mmole) were added to a solution of **5** or **6** and refluxed for 3 h., poured into water, filtered off and crystallized from the proper solvent to give the same product **7a–e**, **8a–e** as two steps of the reaction.

**(E)-2-((2S,3R,4R,5R)-2,3,4,5,6-pentahydroxyhexylidene)-7,8-dihydro-5H,6H-cyclopenta-[4,5]thieno [2,3-d]thiazolo [3,2-a]pyrimidine-3,5(2H)-dione (7a)**

Brown crystals (ethanol), m. p. 207–209 °C, yield: 65%, IR (KBr, cm^−^): 3422 (OH); 2856 (CH); 1662, 1641 (2CO); ^1^H-NMR (DMSO-d_6_, 500 MHZ, δ ppm) 2.37 (m, 2H, H-6^\^, H-6^\\^); 2.34 (m, 1H, H5^\^); 2.83 (t, 2H, CH_2_), 2.5 (t, 1H, H-4^\^); 2.83 (t, 1H, H-3^\^); 2.87 (t,2H, CH_2_); 2.87 (m, 1H, H-2^\^); 3.58 (m, 5H, 5 (OH), D_2_O exchangeable); 6.67 (d, 1H,H-1^\^, J = 7.5 Hz).^13^C-NMR (DMSO-d_6_, 100 MHz, δ ppm): 24.9, 25.6, 31.9 (CH_2_), 63.3, 64.4, 70.9, 72.7, 74.6 (carbon atoms of sugar), 117.6 (C=C=O, pyrimidine), 125.4 (C=C-S, pent), 126.0 (C=C-S, thiazole), 139.4 (C=C=O, pyrimidine), 148.2 (carbon atom of sugar), 155.5 (C=C-S, pent.), 158.3 (N-C=N, pyrimidine), 168.6 (C=O, pyrimidine), 171.3 (N-C=O, thiazole). Anal. Calcd. (%); C_17_H_18_N_2_O_7_S_2_ (426.46): C, 47.88; H, 4.25; N, 6.57; S, 15.04. Found (%); C, 47.90; H, 4.21; N, 6.59; S, 15.1.

**(E)-2-((2R,3R,4R,5R)-2,3,4,5,6-pentahydroxyhexylidene)-7,8-dihydro-5H,6H-cyclopenta [4,5]thieno [2,3-d]thiazolo [3,2-a]pyrimidine-3,5(2H)-dione (7b)**

Brown crystals (ethanol), m. p. 200–202 °C, yield: 52%, IR (KBr, cm^−^): 3434 (OH), 2857 (CH); 1666; 1660 (2 CO). ^1^H-NMR (DMSO-d_6_), 500 MHz, δ ppm: 2.3(m, 2H, CH_2_); 2.49 (t, 2H, CH_2_); 2.5 (t, 2H, CH_2_); 3.05 (m, 1H, H-3^\^); 3.11 (m, 2H, H-6^\^, H-6^\\^); 3.4 (t, 1H, H-4^\^); 3.16 (m, 5H, 5 (OH), D_2_O exchangeable); 3.52 (m, 1H, H-2^\^); 5.96 (d, 1H,H-1^\^, J = 7.5 Hz). ^13^C-NMR (DMSO-d_6_, 100 MHz, δ ppm) 24.9, 25.8 and 31.9 (CH_2_), 63.3, 64.4, 70.9, 72.7, 74.6 (carbon atoms of sugar), 117.6 (C=C, pyrimidine), 125.4 (C= C-S, pent.), 126.0 (C=C-S, thiazole), 139.4 (S-C-N, pent.), 148.2 (carbon atom of sugar), 155.5 (C= C, pyrimidine), 158.3 (N-C=N), 168.6 (C=O, pyrimidine), 171.3 (N-C=O, thiazole). Anal. Calcd. (%) for C_17_H_18_N_2_O_7_S_2_ (426.46): C, 47.88; H, 4.25; N, 6.57; O, 26.26; S, 15.04. Found (%): C, 47.49; H, 4.30; N, 6.48; S, 14.49.

**(E)-2-((2S,3R,4S,5R)-2,3,4,5,6-pentahydroxyhexylidene)-7,8-dihydro-5H,6H-cyclopenta [4,5]thieno [2,3-d]thiazolo [3,2-a]-pyrimidine-3,5(2H)-dione (7c)**

Brown crystals (ethanol), m. p. 334–336 °C, yield: 49%, IR (KBr, cm^−^): 3758 (OH), 2860 (CH); 1668; 1666 (2 CO). ^1^H-NMR (DMSO-d_6_, 500 MHz, δ ppm) 2.34 (m, 1H, H5^\^); 2.48 (t, 2H, CH_2_); 2.49 (t, 2H, CH_2_); 2.83 (m, 2H, H-6^\^, H-6^\\^); 2.85 (m, 3H-3^\^, H-4^\^); 3.56 (m, 5H, 5 (OH), D_2_O exchangeable); 4.2 (dd, 1H, H-2^\^, J = 7.5 Hz); 6.6 (d, 1H,H-1^\^, J = 7.5 Hz). Anal. Calcd. (%): for C_17_H_18_N_2_O_7_S_2_ (426.46): Cal; C, 47.88; H, 4.25; N, 6.57; O, 26.26; S, 15.04. Found (%); C, 47.90; H, 4.0; N, 6.59; S, 15.01.

**(E)-2-((2S,3R,4R)-2,3,4,5-tetrahydroxypentylidene)-7,8-dihydro-5H,6H-cyclopenta [4,5]thieno [2,3-d]thiazolo [3,2-a]-pyrimidine-3,5(2H)-dione (7d)**

Pale brown crystals (ethanol). m. p. 240–242 °C, yield: 66%, IR (KBr, cm^−^): 3152 (OH); 2865 (CH); 1651; 1539 (2 CO); ^1^H-NMR (DMSO-d_6_, 500 MHz, δ ppm); 2.33 (m, 2H, CH_2_); 2.81 (t, 2H, CH_2_); 2.85 (t, 2H, CH_2_); 3.38 (m, 1H, H-4^\^); 3.41 (t, 2H, H-5^\^, H-5^\\^); 3.56 (d, 1H, H-3^\^, J = 5Hz); 3.58 (m, 4H, 4 (OH), D_2_O exchangeable); 5.8 (dd, 1H, H-2^\^, J = 7.5 Hz); 6.68 (d, 1H,H-1^\^, J = 7.5 Hz). ^13^C-NMR (DMSO-d_6_, 100 MHz, δ ppm): 24.9, 25.8 and 31.9 (CH_2_), 63.0, 72.4, 64.5, 76.8 (Sugar carbon atom), 117.6 (C=C, pyrimidine), 125.4 (C=C, pent.), 126.0 (C=C-S, thiazole), 139.4 (C=C, pyrimidine), 148.2 (Sugar carbon atom), 155.5 (C= C, pent.), 158.3 (N-C=N), 168.6 (C=O, pyrimidine), 171.3 (N-C=O, thiazole). Anal. Calcd. (%) for C_16_H_16_N_2_O_6_S_2_ (396.44): Cal; C, 48.47; H, 4.07; N, 7.07; O, 24.21; S, 16.18. Found (%): C, 48.50; H, 4.06; N, 7.05, S, 16.30.

**(E)-2-((2R,3S,4R)-2,3,4,5-tetrahydroxypentylidene)-7,8-dihydro-5H,6H-cyclopenta [4,5]thieno [2,3-d]thiazolo [3,2-a]-pyrimidine-3,5(2H)-dione (7e)**

Brown crystals (ethanol). m. p. 216–217 °C, yield: 69%, IR (KBr, cm^−^): 3423 (OH); 2527 (CH); 1649, 1541 (2 CO); ^1^H-NMR (DMSO, 500 MHz, δ ppm): 2.33 (m, 2 H, CH_2_); 2.35 (t, 2H, CH_2_); 2.49 (t, 2H, CH_2_); 3.38 (m, 1H, H-4^\^); 4.46 (t, 2H, H-5^\^, H-5^\\^); 4.98 (m, 1H, H-3^\^); 3.4 (m, 4H, 4 (OH), D_2_O exchangeable); 4.89 (dd, 1H, H-2^\^, J = 7.5 HZ); 6.62 (d, 1H,H-1^\^, J = 7.5 Hz). Anal. Calcd. (%) for C_16_H_16_N_2_O_6_S_2_ (396.44): C, 48.47; H, 4.07; N, 7.07; O, 24.21; S, 16.18 Found (%): C, 48.39; H, 4.00; N, 7.09; S, 16.21.

**(E)-2-((2S,3R,4R,5R)-2,3,4,5,6-pentahydroxyhexylidene)-6,7,8,9-tetrahydro-5H-benzo [4,5]thieno [2,3-d]thiazolo [3,2-a]-pyrimidine-3,5(2H)-dione (8a)**

Pale yellow crystals (ethanol), m. p. 220–222 °C, yield: 49%, IR (KBr, cm^−^): 3935 (OH), 2854 (CH); 1671, 1540 (2 CO). ^1^H-NMR (DMSO-d_6_, 500 MHz, δ ppm): 1.22 (m, 2H, CH_2_); 1.55 (m, 2H, CH_2_); 2.33 (m, 1H, H5^\^); 2.34 (t, 2H, CH_2_); 2.35 (m, 2H, H-6^\^, H-6^\\^); 2.37 (t, 1H, H-4^\^); 2.52 (t, 1H, H-3^\^); 2.83 (t, 2H, CH_2_); 2.89 (m, 1H, H-2^\^); 3.38 (m, 5H, 5 (OH), D_2_O exchangeable); 6.68 (d, 1H, H-1^\^, J = 7.5 HZ). Anal. Calcd. (%) For C_18_H_20_N_2_O_7_S_2_ (440.49): C, 49.08; H, 4.58; N, 6.36; O, 25.43; S, 14.56. Found (%): C, 49.00; H, 4.55; N, 6.40; S, 25.49.

**(E)-2-((2R,3R,4R,5R)-2,3,4,5,6-pentahydroxyhexylidene)-6,7,8,9-tetrahydro-5H-benzo-[4,5]thieno [2,3-d]thiazolo [3,2-a]pyrimidine-3,5(2H)-dione (8b)**

Brown crystals (ethanol), m. p. 243–244 °C, yield: 65%, IR (KBr, cm^−^) 3419 (OH), 2853 (CH); 1671, 1530 (2 CO); ^1^H-NMR (DMSO-d_6_, 500 MHZ, δ ppm): 1.79 (m, 2H, CH_2_); 1.8 (m, 2H, CH_2_); 2.33 (m, 1H, H5^\^); 2.72 (t, 2H, CH_2_); 2.83 (t, 2H, CH_2_); 3.11 (m, 2H, H-6^\^, H-6^\\^); 3.40 (t, 1H, H-4^\^); 3.52 (m, 5H, 5 (OH), D_2_O exchangeable); 3.68 (m, 1H, H-2^\^); 3.75 (t, 1H, H-3^\^); 6.20 (d, 1H, H-1^\^, J = 7.5 Hz). ^13^C-NMR (DMSO-d_6_, 100 MHz, δ ppm): 23.0, 25.4 and 24.5 (CH_2_), 63.3, 64.4, 70.9, 72.7 and 74.6 (carbon atoms of sugar), 117.6 (C=C, pyrimidine), 125.4 (C= C, hex.), 126.0 (C=C-S, thiazole), 139.4 (C=C, hex.), 148.2 (carbon atom of sugar), 155.5 (C=C, pyrimidine), 158.3 (N-C=N), 168.6 (C=O, pyrimidine), 171.3 (N-C=O, thiazole). Anal. Calcd. (%) For C_18_H_20_N_2_O_7_S_2_ (440.49): C, 49.08; H, 4.58; N, 6.36; O, 25.43; S, 14.56. Found (%): C, 49.10; H, 4.55; N, 6.29.

**(E)-2-((2S,3R,4S,5R)-2,3,4,5,6-pentahydroxyhexylidene)-6,7,8,9-tetrahydro-5H-benzo [4,5]-thieno [2,3-d]thiazolo [3,2-a]-pyrimidine-3,5(2H)-dione (8c)**

Gray crystals (ethanol), m. p. 209–211 °C, yield: 55%, IR (KBr, cm^−^) 3419 (OH), 2853 (CH); 1671, 1530 (2 CO); ^1^H-NMR (DMSO-d_6_, 500 MHz, δ ppm): 1.74 (m, 2H, CH_2_); 1.94 (m, 2H, CH_2_); 2.33 (m, 1H, H5^\^); 2.49 (t, 2H, CH_2_); 2.5 (t, 2H, CH_2_); 2.83 (m, 2H, H-6^\^, H-6^\\^); 2.85 (m, 3H,H-3^\^, H-4^\^, H-5^\^); 3.56 (m, 5H, 5 (OH), D_2_O exchangeable); 4.2 (dd, 1H, H-2^\^, J = 7.5 Hz); 6.6 (d, 1H,H-1^\^, J = 7.5 Hz). Anal. Calcd. (%) for C_18_H_20_N_2_O_7_S_2_ (440.49): C, 49.08; H, 4.58; N, 6.36; O, 25.43; S, 14.56. Found: (%) C, 48.99; H, 4.44; N, 6.46; 25.41; S, 14.46.

**(E)-2-((2S,3R,4R)-2,3,4,5-tetrahydroxypentylidene)-6,7,8,9-tetrahydro-5H-benzo [4,5]thieno [2,3-d]thiazolo [3,2-a]-pyrimidine-3,5(2H)-dione (8d)**

Gray crystals (ethanol), m. p. 215–217 °C, yield: 70%, IR (KBr, cm^−^) 3457 (OH), 2457 (CH); 1637, 1531,1542 (2 CO); ^1^H-NMR (DMSO-d_6_, 500 MHz, δ ppm): 1.78 (m, 2H, CH_2_); 1.94 (m, 2H, CH_2_); 1.8 (t, 2H, CH_2_); 3.95 (t, 2H, CH_2_); 3.38 (m, 1H, H-4^\^); 3.39 (t, 2H, H-5^\^, H-5^\\^); 3.49 (d, 1H, H-3^\^, J = 5Hz); 3.56 (m, 4H, 4 (OH), D_2_O exchangeable); 5.98 (dd, 1H, H-2^\^, J = 7.5 Hz); 6.7 (d, 1H,H-1^\^, J = 7.5 Hz). ^13^C-NMR (DMSO-d_6_, 100 MHz, δ ppm): 23.0, 23.4 and 24.5 (CH_2_), 63.0, 72.4, 64.5, 76.8 (sugar carbon atom), 117.6 (C=C, pyrimidine), 125.4 (C=C, hex.), 126.0 (C=C-S, thiazole), 139.4 (C=C, hex.), 148.2 (sugar carbon atom), 155.5 (C=C, pyrimidine.), 158.3 (N=C-N, pyrimidine), 168.6 (C=O, pyrimidine), 171.3 (N-C=O, thiazole). Anal. Calcd. (%) for C_17_H_18_N_2_O_6_S_2_ (410.46): C, 49.74; H, 4.42; N, 6.82; O, 23.39; S, 15.62 Found (%): C, 49.60; H, 4.39; N, 6.79; S, 1564.

**(E)-2-((2R,3S,4R)-2,3,4,5-tetrahydroxypentylidene)-6,7,8,9-tetrahydro-5H-benzo [4,5]thieno [2,3-d]thiazolo [3,2-a]pyrimidine-3,5(2H)-dione (8e)**

Gray crystals (ethanol), m. p. 233–235 °C, yield: 43%, IR (KBr, cm^−3^): 3423 (OH); 2854 (CH); 1669, 1529 (2 CO); ^1^H-NMR (DMSO-d_6_, 500 MHz, δ ppm) 1.77 (m, 2H, CH_2_); 1.79 (m, 2H, CH_2_); 2.5 (t, 2H, CH_2_); 2,67 (t, 2H, CH_2_), 3.38 (m, 1H, H-4^\^); 4.5 (t, 2H, H-5^\^, H-5^\\^); 4.00 (m, 1H, H-3^\^); 3.41 (m, 4H, 4 (OH), D_2_O exchangeable); 4.9 (dd, 1H, H-2^\^, J = 7.5 Hz); 6.60 (d, 1H,H-1^\^, J = 7.5 Hz). Anal. Calcd. (%) for C_17_H_18_N_2_O_6_S_2_ (410.46): C, 49.74; H, 4.42; N, 6.82; O, 23.39; S, 15.62, Found (%): C, 49.81; H, 4.39; N, 6.79; S, 15.55.

### 3.2. In Vitro Biological Activities

All in vitro biological activities were evaluated for the synthetic compounds at a fixed concentration of 10 µg/mL, and each experiment was conducted in triplicate.

#### 3.2.1. Antioxidant and Scavenging Activity

The antioxidant activity was assessed using the iron reducing power (IRP) [74] and the total antioxidant capacity (TAC) [75]. Ascorbic acid served as a standard to calculate the inhibition percentages (%) and the median inhibitory concentration (IC_50_) of each tested compound against the following radicals: nitric oxide (NO) [76], hydroxyl (OH) [77], hydrogen peroxide (H_2_O_2_) [78], ABTS [79], and DPPH [80].

#### 3.2.2. Anti-Diabetic Activity

The anti-diabetic activity was determined using acarbose as a reference drug. The IC_50_ and inhibition percentages (Inhib. %) of the enzymes α-amylase [81] and α-glucosidase [82] were calculated.

#### 3.2.3. Anti-Alzheimer’s Activity

The anti-Alzheimer’s activity was assessed using donepezil as a reference drug. The inhibition percentage and IC_50_ of the acetylcholinesterase (AChE) enzyme were measured using the Ellman method [83].

#### 3.2.4. Anti-Arthritic Activity

Anti-arthritic activity was evaluated using diclofenac sodium as the reference drug. The inhibition percentage and IC_50_ were determined for protein denaturation [84] and the proteinase enzyme [85].

### 3.3. Molecular Docking Study

The 2D structures of thieno [2,3-d]thiazolo [3,2-*a*]pyrimidine glycosides **8b** and **8e** were drawn through ChemDraw. The protonated 3D was employed using standard bond lengths and angles, using Molecular Operating Environment (MOE-Dock) software version 2024.0601 [86,87,88,89]. Then, the geometry optimization and energy minimization were applied to get the Conf Search module in MOE, followed by saving of the moe file for upcoming docking process. The co-crystallized structures of α-amylase, α-glucosidase with their native ligand, acarbose, were downloaded from the protein data bank (PDB codes: 1B2Y and 3WY1, respectively). All minimizations were performed using MOE until an RMSD gradient of 0.05 kcal∙mol^−1^Å^−1^ with MMFF94x force field and the partial charges were automatically calculated. Preparation of the enzymes’ structures were done for molecular docking using Protonate 3D protocol with the default options in MOE. London dG scoring function and Triangle Matcher placement method were used in the docking protocol. Initially, the validation of the docking processes was established by docking the native ligand, followed by docking the derivatives **8b** and **8e** within the ATP-binding sites after eliminating the co-crystallized ligand.

### 3.4. Molecular Dynamics (MD) Simulations

The incorporation of Molecular Dynamics (MD) simulations in the study of biological systems facilitates the examination of atomic and molecular motion that is not readily accessible through alternative methods. The analysis derived from this simulation offers a complex view of the dynamic evolution of biological systems, including conformational changes and molecular associations [90].

The molecular dynamics simulations of all systems were conducted with the GPU version of the PMEMD engine included in the AMBER 18 package [91]. The partial atomic charge for each chemical was determined using ANTECHAMBER’s General Amber Force Field (GAFF) methodology [92]. The Leap module of the AMBER 18 package implicitly solvated any system within an orthorhombic box of TIP3P water molecules, maintaining a distance of 10 Å from any box edge. The Leap module was employed to neutralize each system by integrating Na^+^ and Cl^−^ counter ions.

An initial minimization of 2000 steps for each system was performed with a 500 kcal/mol imposed restraint potential, succeeded by a 1000-step complete minimization utilizing the conjugate gradient algorithm without restraints. In the MD simulation, each system was incrementally heated from 0 K to 300 K over a duration of 500 ps, maintaining uniformity in atom count and volume across all systems. The system’s solutes were exposed to a 10 kcal/mol potential harmonic limit and a 1 ps collision frequency. Subsequently, each system was subjected to heating and equilibrated for 500 ps at a constant temperature of 300 K.

To replicate an isobaric-isothermal (NPT) ensemble, the atom count and pressure in each system during production simulations were held constant, with the pressure regulated at 1 bar utilizing the Berendsen barostat [93]. Each system underwent molecular dynamics simulation for 20 nanoseconds. The SHAKE algorithm was employed to restrict the hydrogen bond atoms in each simulation. Each simulation employed a 2 fs timestep and integrated a single-precision floating-point model. The simulations employed an isobaric-isothermal ensemble (NPT) with randomized seeding, maintaining a constant pressure of 1 bar, a pressure-coupling constant of 2 ps, a temperature of 300 K, and a Langevin thermostat featuring a collision frequency of 1 ps.

#### Post-MD Analysis

After saving the trajectories obtained by MD simulations every 1 ps, the trajectories were analyzed using the AMBER18 suite’s CPPTRAJ [90] module. The Origin [91] data analysis program was used to create all graphs and visualizations.

## 4. Conclusions

In this study, previously synthesized glycosylated thienopyrimidinone derivatives (**7a–e** and **8a–e**) were evaluated for their antioxidant, anti-Alzheimer, anti-arthritic, and anti-diabetic activities. Among these, compound **8e** demonstrated superior performance in antioxidant, anti-arthritic, and anti-diabetic assays, while compound **8c** exhibited notable AChE inhibitory activity. The incorporation of sugar moieties, particularly arabinose and mannose, was found to enhance the biological profiles of the parent scaffold, supporting their potential as multifunctional therapeutic leads.

The current investigation is limited to in vitro assays and molecular docking simulations. No experiments were conducted using neuronal or astrocytic models, and critical parameters such as cytotoxicity, neuronal marker expression, and functional outcomes in neural cells were not assessed. As such, the proposed neuroprotective effects remain hypothetical and require further validation through in vivo studies and mechanistic exploration.

Future work should focus on in vivo validation, expanded biological screening, and elucidation of the molecular mechanisms underlying the observed activities. Additionally, the synthesis of new sugar-conjugated analogs may refine structure-activity relationship (SAR) insights and support the rational development of next-generation thienopyrimidinone-based therapeutics.

## Figures and Tables

**Table 1:** Total antioxidant capacity (TAC) and iron-reducing power (IRP) of the compounds (**7a–8e**) compared with ascorbic acid.

No.	TAC (mg Gallic Acid/g)	IRP (µg/mL)	DPPH (%)	ABTS (%)	NO (%)	OH (%)	H_2_O_2_ (%)
**7a**	17.44 ± 0.22	10.29 ± 0.22	14.04 ± 0.22	17.19 ± 0.22	8.29 ± 0.22	7.21 ± 0.19	8.07 ± 0.21
**7b**	21.59 ± 0.25	14.44 ± 0.25	18.19 ± 0.25	21.34 ± 0.25	12.44 ± 0.25	10.82 ± 0.21	12.12 ± 0.24
**7c**	20.40 ± 0.26	13.25 ± 0.26	17.00 ± 0.26	20.15 ± 0.26	11.25 ± 0.26	9.78 ± 0.22	10.96 ± 0.25
**7d**	20.09 ± 0.23	12.94 ± 0.23	16.69 ± 0.23	19.84 ± 0.23	10.94 ± 0.23	9.51 ± 0.20	10.65 ± 0.22
**7e**	18.77 ± 0.21	11.62 ± 0.21	15.37 ± 0.21	18.52 ± 0.21	9.62 ± 0.21	8.37 ± 0.19	9.37 ± 0.21
**8a**	13.95 ± 0.18	6.80 ± 0.18	10.55 ± 0.18	13.70 ± 0.18	4.80 ± 0.18	4.17 ± 0.15	4.67 ± 0.17
**8b**	68.01 ± 0.78	60.86 ± 0.78	64.61 ± 0.78	67.76 ± 0.78	58.86 ± 0.78	51.18 ± 0.68	57.32 ± 0.76
**8c**	15.90 ± 0.20	8.75 ± 0.20	12.50 ± 0.20	15.65 ± 0.20	6.75 ± 0.20	5.87 ± 0.17	6.58 ± 0.20
**8d**	17.02 ± 0.21	9.87 ± 0.21	13.62 ± 0.21	16.77 ± 0.21	7.87 ± 0.21	6.84 ± 0.19	7.66 ± 0.21
**8e**	68.69 ± 0.79	61.54 ± 0.79	65.29 ± 0.79	68.44 ± 0.79	59.54 ± 0.79	51.77 ± 0.68	57.99 ± 0.76
**STD**	78.44 ± 0.19	67.49 ± 0.17	71.24 ± 0.17	74.39 ± 0.17	65.49 ± 0.17	56.95 ± 0.14	63.78 ± 0.16

The values were calculated from n = 3/extract and given as mean ± SE.

**Table 2:** Radical scavenging activity (IC_50_, µg/mL) of the compounds (**7a–8e**) in DPPH, ABTS, NO, OH, and H_2_O_2_ assays compared with ascorbic acid.

Sample	DPPH	ABTS	NO	OH	H_2_O_2_
**7a**	27.67 ± 0.29	18.83 ± 0.22	48.89 ± 0.78	65.77 ± 2.12	73.10 ± 1.67
**7b**	21.36 ± 0.44	15.17 ± 0.15	32.58 ± 0.96	43.75 ± 0.52	48.67 ± 0.85
**7c**	22.84 ± 0.23	16.06 ± 0.19	36.00 ± 0.45	48.42 ± 1.40	53.83 ± 1.05
**7d**	23.28 ± 0.49	16.31 ± 0.16	37.06 ± 1.13	49.77 ± 0.65	55.36 ± 1.03
**7e**	25.27 ± 0.54	17.47 ± 0.17	42.12 ± 1.33	56.57 ± 0.81	62.92 ± 1.25
**8a**	36.82 ± 0.42	23.63 ± 0.28	84.51 ± 2.12	113.71 ± 4.73	126.37 ± 4.09
**8b**	6.01 ± 0.12	4.78 ± 0.05	6.88 ± 0.16	9.24 ± 0.05	10.28 ± 0.12
**8c**	31.07 ± 0.34	20.68 ± 0.24	60.02 ± 1.12	80.74 ± 2.84	89.75 ± 2.32
**8d**	28.53 ± 0.30	19.31 ± 0.23	51.52 ± 0.85	69.29 ± 2.28	77.03 ± 1.82
**8e**	5.95 ± 0.12	4.73 ± 0.05	6.80 ± 0.16	9.14 ± 0.05	10.17 ± 0.12
**STD**	5.45 ± 0.06	4.35 ± 0.04	6.18 ± 0.09	8.31 ± 0.05	9.24 ± 0.04

The values were calculated from n = 3/extract and given as mean ± SE.

**Table 3:** Anti-Alzheimer’s and anti-arthritic activities of the compounds (**7a–8e**).

Sample	AChE Inhibition (%)	AChE IC_50_ (µg/mL)	Protein Denaturation Inhibition (%)	Proteinase Inhibition (%)	Proteinase IC_50_ (µg/mL)
**7a**	5.75 ± 0.18	49.93 ± 1.96	11.62 ± 0.18	7.45 ± 0.18	62.33 ± 1.77
**7b**	5.14 ± 0.16	55.92 ± 2.20	15.06 ± 0.20	10.89 ± 0.20	42.61 ± 0.37
**7c**	5.87 ± 0.19	48.95 ± 1.93	14.08 ± 0.21	9.91 ± 0.21	46.88 ± 1.21
**7d**	4.40 ± 0.14	65.33 ± 2.57	13.82 ± 0.19	9.65 ± 0.19	48.10 ± 0.46
**7e**	4.11 ± 0.13	69.90 ± 2.75	12.73 ± 0.18	8.56 ± 0.18	54.22 ± 0.58
**8a**	5.81 ± 0.18	49.44 ± 1.95	8.74 ± 0.15	4.57 ± 0.15	101.84 ± 3.59
**8b**	5.19 ± 0.16	55.37 ± 2.18	11.80 ± 0.19	7.63 ± 0.19	65.93 ± 2.02
**8c**	5.93 ± 0.19	48.46 ± 1.91	10.35 ± 0.17	6.18 ± 0.17	75.16 ± 2.30
**8d**	5.61 ± 0.18	51.23 ± 2.02	11.28 ± 0.18	7.11 ± 0.18	65.40 ± 1.90
**8e**	5.24 ± 0.17	54.82 ± 2.16	54.07 ± 0.65	50.90 ± 0.65	9.08 ± 0.11
**STD**	54.46 ± 0.16	5.26 ± 0.05	60.74 ± 0.14	56.57 ± 0.14	8.20 ± 0.06

The values were calculated from n = 3/extract and given as mean ± SE. STD (Donepezil/Diclofenac).

**Table 4:** Anti-diabetic activities of synthetic compounds (inhibition % at 20 µg/mL and IC_50_ values).

	Anti-Diabetic Activity			
Sample	α-Amylase	α-Glucosidase	α-Amylase	α-Glucosidase
	Inhibition (%)		IC_50_ (µg/mL)	
**7a**	13.37 ± 0.21	31.25 ± 0.65	6.62 ± 0.21	31.92 ± 0.89
**7b**	17.32 ± 0.24	24.10 ± 0.08	10.57 ± 0.24	19.96 ± 0.34
**7c**	16.19 ± 0.24	25.80 ± 0.52	9.44 ± 0.24	22.37 ± 0.53
**7d**	15.89 ± 0.22	26.27 ± 0.09	9.14 ± 0.22	23.09 ± 0.41
**7e**	14.64 ± 0.20	28.51 ± 0.11	7.89 ± 0.20	26.75 ± 0.52
**8a**	10.05 ± 0.17	41.58 ± 0.89	3.30 ± 0.17	64.26 ± 2.84
**8b**	61.53 ± 0.74	6.78 ± 0.02	54.78 ± 0.74	3.85 ± 0.05
**8c**	11.91 ± 0.19	35.08 ± 0.74	5.16 ± 0.19	41.00 ± 1.33
**8d**	12.97 ± 0.20	32.21 ± 0.67	6.22 ± 0.20	33.99 ± 0.98
**8e**	62.18 ± 0.75	6.71 ± 0.02	55.43 ± 0.75	3.81 ± 0.05
**(Acarbose)**	69.85 ± 0.16	5.98 ± 0.05	63.10 ± 0.16	3.34 ± 0.05

## Data Availability

The original contributions presented in this study are included in the article. Further inquiries can be directed to the corresponding author.
